# Rare pathological types of gallbladder cancer: Cases report

**DOI:** 10.1097/MD.0000000000048350

**Published:** 2026-05-01

**Authors:** Hao Wang, Zepu Wang, Yang Zhao, Yaqi Xiang, Jun Li, Hongfang Tuo

**Affiliations:** a Graduate School of Hebei Medical University, Shijiazhuang, Hebei, China; b Department of Hepatobiliary and Pancreatic Surgery, Hebei General Hospital, Shijiazhuang, Hebei, China; c Hebei North University, Zhangjiakou, Hebei, China.

**Keywords:** gallbladder cancer, gallbladder carcinosarcoma, gallbladder sarcomatoid carcinoma

## Abstract

**Rationale:**

Gallbladder cancer is one of the most aggressive malignancies in the biliary system, and there is currently no internationally recognized standardized treatment protocol. Reports on the management of rare pathological types of gallbladder cancer are particularly limited. We present 2 cases of gallbladder cancer with rare pathological subtypes and their diagnostic and therapeutic processes, aiming to further enhance clinicians’ understanding of these uncommon entities.

**Patient concerns:**

This report describes 2 cases of gallbladder cancer, both presenting with abdominal discomfort, raising concerns about the nature of the lesion and effective treatment strategies.

**Diagnoses:**

Postoperative pathology confirmed one case as gallbladder sarcomatoid carcinoma and the other as gallbladder carcinosarcoma, both of which are clinically rare types.

**Interventions:**

Both patients underwent laparoscopic cholecystectomy at our institution, with subsequent pathological and immunohistochemical examination.

**Outcomes:**

As of the end of follow-up (November 30, 2025), both patients recovered well postoperatively.

**Lessons:**

Rare types of gallbladder cancer lack specific clinical manifestations, making early screening difficult. Most patients are diagnosed at an advanced stage. Surgical intervention should be performed as early as possible upon discovery.

## 1. Introduction

Gallbladder cancer is a common malignant tumor of the biliary system. Its global incidence shows significant geographical variation, with higher rates in populations such as Chile and India. The etiological factors remain unclear, with chronic inflammatory stimulation from conditions like gallstones or bacterial infection believed to cause mucosal damage, promoting progression from gallbladder mucosa to dysplasia.^[1]^ Approximately half of the patients are incidentally found to have a gallbladder mass during physical examination, with gallbladder cancer revealed by postoperative pathology. Treatment for gallbladder cancer is not yet standardized, and management for its rare types is even scarcer.

## 2. Case presentation

### 2.1. Case 1

A 65-year-old male was admitted on July 29, 2024, due to “intermittent distension and discomfort in the right upper quadrant for 2 months.” The patient had a healthy past medical history, with no other diseases or long-term medication use. Physical examination revealed no positive signs, and no jaundice was observed in the skin or sclera. Preoperative tests: Tumor markers: Ferritin: 144.5 ng/mL, CA19-9: 12.9 U/mL, carcinoembryonic antigen (CEA): 3.36 ng/mL, alpha-fetoprotein (AFP): 3.68 ng/mL; other laboratory tests and tumor markers were within normal limits. Subsequent contrast-enhanced computed tomography (CT) of the liver, gallbladder, and pancreas indicated a gallbladder mass (Fig. 1A), which was further confirmed by ultrasonography. After excluding surgical contraindications via relevant tests, the patient underwent fluorescence-guided laparoscopic radical cholecystectomy under general anesthesia on August 2, 2024. Preoperative contrast-enhanced CT and ultrasound suggested a gallbladder mass with suspected malignancy. Intraoperatively, the tumor was localized to the gallbladder fundus without obvious invasion of the liver parenchyma or surrounding structures. Intraoperative frozen section examination revealed diffuse proliferation of atypical cells within the mucosa, consistent with malignancy. Based on intraoperative findings and our institutional protocol for suspected gallbladder cancer, fluorescence-guided laparoscopic radical cholecystectomy was performed, including cholecystectomy, wedge resection of the gallbladder bed, and dissection of group 12 lymph nodes. The resected specimen revealed the gallbladder filled with sandy stones and a polypoid lesion near the fundus. Postoperative pathology report: (Gallbladder mass) Malignant tumor, consistent with sarcomatoid carcinoma based on immunohistochemical staining, infiltrating the muscularis propria. No definite lymphovascular invasion or perineural invasion was identified. Immunohistochemistry: cytokeratin pan (CKpan) (−), epithelial membrane antigen (EMA) (focal+), Vimentin (+), tumor protein 53 (scattered+), Ki-67 antigen (approximately 60%+), CEA (−), cluster of differentiation 34 (CD34) (−), S100 calcium-binding protein (S100) (−), spinal muscular atrophy (focal+), Desmin (−), cluster of differentiation 56 (+), synaptophysin (−). To further characterize the tumor and exclude other spindle cell malignancies, additional epithelial markers were evaluated: cytokeratin 7 (−), cytokeratin 19 (−), and AE1/AE3 (−); (Gallbladder margin) No carcinoma identified; (Liver tissue from gallbladder bed) No carcinoma identified. Hepatocytes showed granular degeneration, focal steatosis, localized cholestasis, and portal tract infiltration by numerous lymphocytes (Fig 2A: H&E, original magnification 10 × 10, Fig. 2B: H&E, original magnification 20 × 10). Postoperative diagnosis: Gallbladder cancer (sarcomatoid carcinoma, T1bN0M0, Stage I). A total of 4 lymph nodes from the group 12 station were retrieved, all negative for metastasis. The patient was discharged on the 5th postoperative day. He subsequently received 2 cycles of adjuvant chemotherapy with gemcitabine hydrochloride (1.4 g) and capecitabine (1 g twice daily). Follow-up examinations at 1, 3, and 6 months postoperatively, including ultrasonography and contrast-enhanced magnetic resonance imaging (MRI) of the hepatobiliary system, showed no evidence of tumor recurrence or distant metastasis. Multiple outpatient reviews of tumor markers and other tests revealed no abnormalities. The patient is currently continuing oral chemotherapy and remains under follow-up.

**Figure 1.**
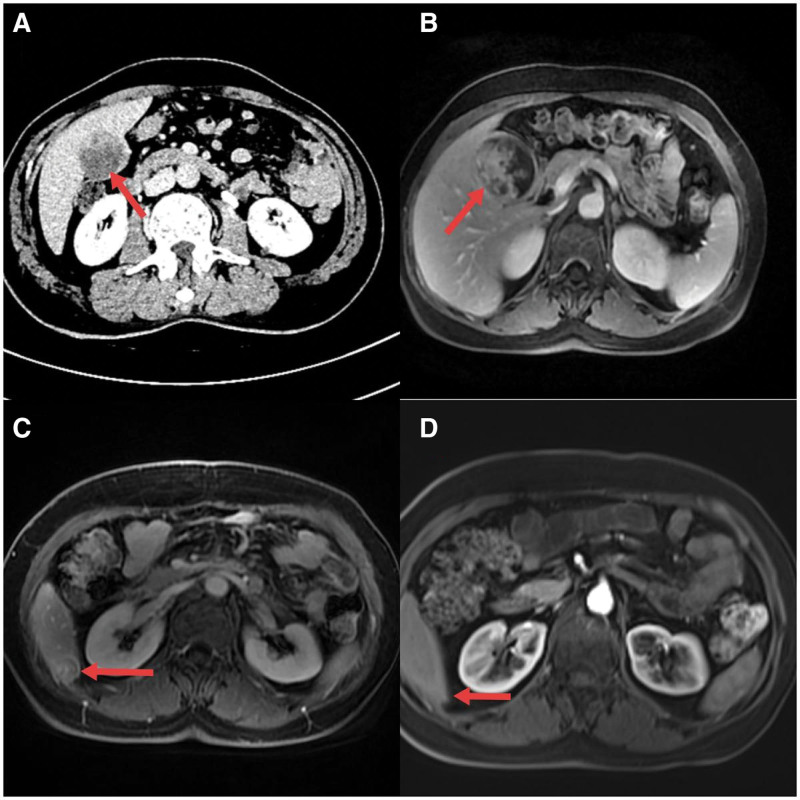
Imaging findings of gallbladder cancer. (A) Preoperative contrast-enhanced CT scan of gallbladder sarcomatoid carcinoma (Case 1) showing a polypoid mass in the gallbladder fundus (arrow). (B) Preoperative MRI of gallbladder carcinosarcoma (Case 2) revealing a heterogeneous mass with irregular enhancement (arrow). (C) Postoperative MRI at 6 mo (Case 2) demonstrating a subcapsular metastatic nodule at the inferior liver edge (arrow). (D) MRI after 4 cycles of toripalimab-based therapy (Case 2) showing significant regression of the metastatic lesion (arrow). CT = computed tomography, MRI = magnetic resonance imaging.

**Figure 2.**
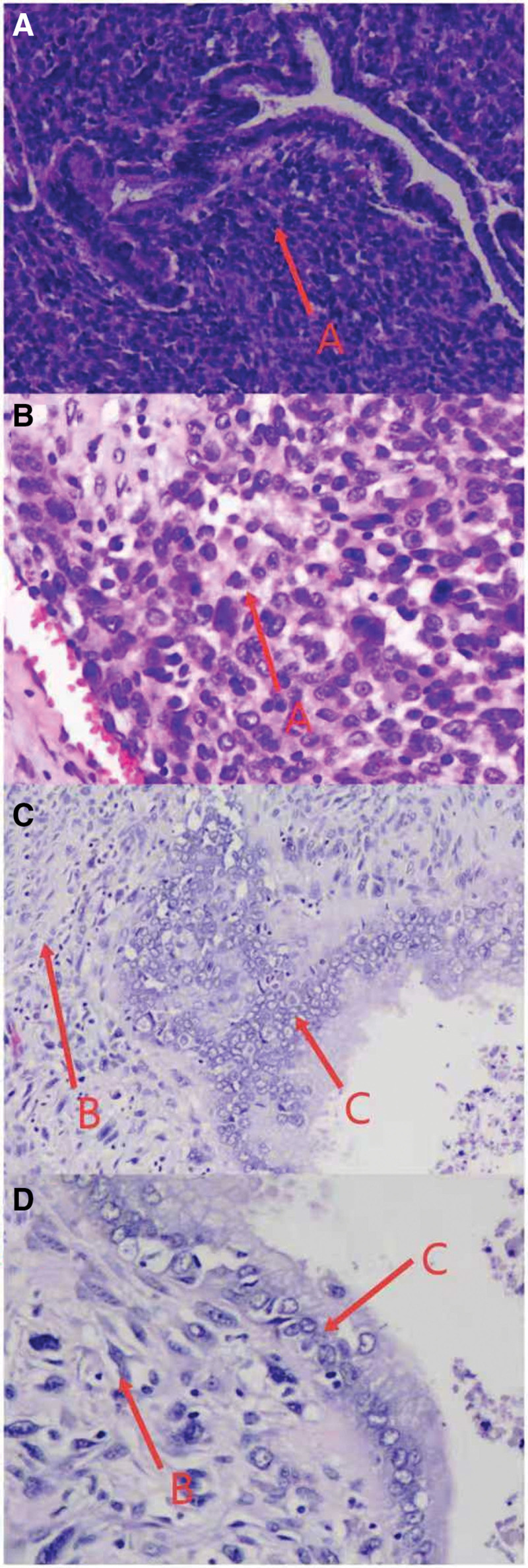
Histopathological findings. (A) Gallbladder sarcomatoid carcinoma (Case 1): spindle-shaped tumor cells arranged in fascicles (component A, arrow) (H&E, original magnification 10 × 10). (B) Higher magnification (20×) of gallbladder sarcomatoid carcinoma (Case 1) showing pleomorphic spindle cells (component consistent with panel a, arrow) (H&E, original magnification 20×). (C) Gallbladder carcinosarcoma (Case 2): biphasic tumor composed of fibrosarcoma (component B, arrow) and well-differentiated adenocarcinoma (component C, arrowhead) (H&E, original magnification 10 × 10). (D) Higher magnification (20×) of gallbladder carcinosarcoma (Case 2) showing atypical spindle cells in the sarcomatous area (component consistent with panel c, arrow) (H&E, original magnification 20×).

### 2.2. Case 2

A 52-year-old female was admitted on August 19, 2024, due to “distension and discomfort in the upper abdomen for half a month.” The patient had a healthy past medical history, with no other diseases or long-term medication use. Physical examination revealed no positive signs, and no jaundice was observed. Preoperative tests: Tumor markers: Ferritin: 280.4 ng/mL, CA19-9: 12.23 U/mL, CEA: 1.89 ng/mL, AFP: 3.1 ng/mL; other tests were normal. Subsequent contrast-enhanced MRI of the liver, gallbladder, and spleen indicated a gallbladder mass (Fig. 1A). On August 22, 2024, the patient underwent fluorescence-guided laparoscopic radical cholecystectomy under general anesthesia. Preoperative MRI suggested a gallbladder mass with suspected malignancy. Intraoperatively, the tumor was found to be adherent to the liver bed, raising suspicion of hepatic involvement. Therefore, fluorescence-guided laparoscopic radical cholecystectomy with resection of liver segments IVb and V was performed to achieve an R0 margin. The specimen revealed the gallbladder filled with a soft tissue tumor connected to the hepatic bed surface, without invasion into the liver parenchyma. Postoperative pathological diagnosis: Gallbladder: Carcinosarcoma (fibrosarcoma + well-differentiated adenocarcinoma), infiltrating the perimuscular soft tissue (without full-thickness penetration of the wall). No lymphovascular invasion or perineural invasion was seen. (Cystic duct margin) No tumor identified. A total of 6 lymph nodes from stations 8, 12, and 13 were dissected, all negative for metastatic tumor. Immunohistochemistry: Sarcomatous area (fibrosarcoma): CKpan (−), Vimentin (+), spinal muscular atrophy (focal+), Ki-67 antigen (approximately 40%+ in active areas), Desmin (−), S100 (−), CD34 (vascular+), discovered on GIST-1 (−), CD117 (−), anaplastic lymphoma kinase (−); Carcinomatous area (well-differentiated adenocarcinoma): CKpan (epithelial+), EMA (epithelial+); Gallbladder body: Tubular adenoma with high-grade intraepithelial neoplasia; Partial liver tissue: No tumor involvement. Partial hepatocyte steatosis and mild inflammatory cell infiltration in portal tracts (G2S0) (Fig 2C: H&E, original magnification 10 × 10, Fig. 2D: H&E, original magnification 20 × 10). Postoperative diagnosis: Gallbladder carcinosarcoma (T2bN0M0, Stage IIA). The patient was discharged on the 5th postoperative day. She received 3 cycles of adjuvant chemotherapy with gemcitabine hydrochloride (1.4 g). Follow-up at 1 and 3 months showed no recurrence. However, at 6 months postoperatively, contrast-enhanced MRI revealed a subcapsular nodule with abnormal signal at the inferior liver edge, suggestive of metastasis (Fig. 1C). PET-CT indicated “status post gallbladder carcinosarcoma resection” with no abnormal mass or hypermetabolism in the surgical bed, but multiple nodular and patchy peritoneal thickenings with increased metabolism, most prominent at the inferior liver edge, bilateral mid-to-lower abdomen, and pelvis, consistent with multifocal peritoneal metastasis (Fig 3C, D). Tumor markers: Ferritin: 614.3 ng/mL, CA19-9: 11.2 U/mL, CEA: 6.131 ng/mL, AFP: 6.887 ng/mL. Other tests were normal. Following multidisciplinary discussion, the patient was started on toripalimab (240 mg) + oxaliplatin (150 mg) + oral lenvatinib (8 mg) + oral capecitabine (1.5 g), with cycles repeated every 4 weeks. After 4 cycles, repeat MRI showed significant lesion reduction (Fig. 1D). During the 5th cycle, PET-CT indicated “status post resection and comprehensive treatment,” with no recurrence in the surgical bed. The previously noted multifocal peritoneal metastases had resolved to normal structure and metabolism. A slightly hypermetabolic lymph node above the right diaphragm showed decreased metabolism and reduced size (Fig. 3A, B). Serial tumor markers showed improvement. The most recent results were: Ferritin: 205.6 ng/mL, CA19-9: 7.8 U/mL, CEA: 2.315 ng/mL, AFP: 7.884 ng/mL. The patient’s condition was considered in remission. She experienced no adverse reactions. As of the last follow-up, she had completed 7 cycles of this regimen and remains under follow-up.

**Figure 3.**
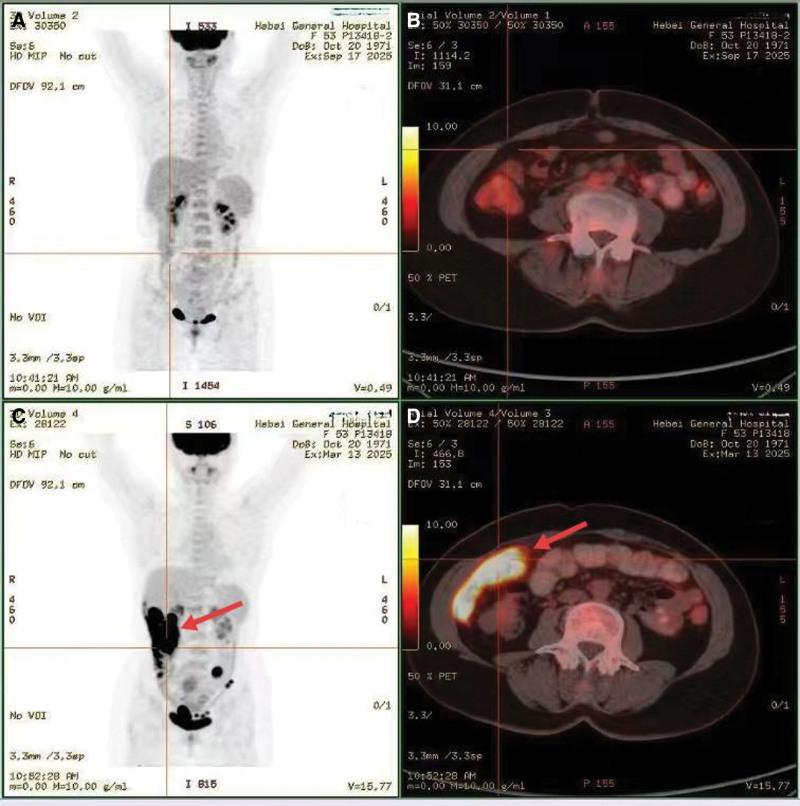
Representative PET-CT images of gallbladder carcinosarcoma (Case 2). (A, B) After 5 cycles of toripalimab-based therapy, PET-CT shows no abnormal hypermetabolism in the surgical bed and resolution of prior peritoneal metastases. (C, D) Postoperative PET-CT at the time of recurrence (6 mo) demonstrating multiple hypermetabolic peritoneal lesions (arrows), consistent with multifocal peritoneal metastasis. CT = computed tomography.

## 3. Discussion

This report presents 2 cases of uncommon pathological subtypes of gallbladder cancer. Gallbladder sarcomatoid carcinoma is a highly malignant and extremely rare tumor.^[2,3]^ Morphologically resembling spindle cell sarcoma, it actually originates from epithelial tissue, exhibiting differentiation of cancer cells towards a sarcomatous direction with spindle cell variation, but it is fundamentally a carcinoma.^[4]^ The pathogenesis of gallbladder sarcomatoid carcinoma remains unclear. Some research suggests its development may be closely related to epithelial-mesenchymal transition, where activation of this pathway plays a significant role in the transformation of epithelial components into sarcomatoid cells.^[5,6]^

The diagnosis of sarcomatoid carcinoma in Case 1 was based on the co-expression of epithelial (EMA focal+) and mesenchymal (vimentin diffuse+) markers, together with spindle cell morphology. Although complete loss of cytokeratin expression (CKpan, cytokeratin 7, cytokeratin 19, AE1/AE3 all negative) is unusual, it is recognized in poorly differentiated sarcomatoid carcinomas due to epithelial-mesenchymal transition.^[5,6]^ This immunoprofile helps distinguish sarcomatoid carcinoma from pure sarcomas such as undifferentiated pleomorphic sarcoma or leiomyosarcoma, which would typically lack any epithelial marker expression and show diffuse positivity for vimentin alone. The negative results for desmin, S100, and CD34 further excluded specific sarcomatous differentiation. Thus, the diagnosis of sarcomatoid carcinoma is firmly established despite the atypical cytokeratin negativity.Gallbladder carcinosarcoma is an exceedingly rare malignant tumor of the gallbladder, composed of both malignant epithelial and mesenchymal cells.^[7]^ Based on the mesenchymal component, it can be classified into 2 types: one containing specific sarcomatous elements like chondrosarcoma, osteosarcoma, or rhabdomyosarcoma; and another where the malignant mesenchymal component consists of markedly atypical spindle cells of uncertain histological origin, termed undifferentiated sarcoma. There is typically no transition between the carcinomatous and sarcomatous components.^[8]^ Its pathogenesis remains unclear, but it is recognized as one of the most aggressive biliary tract malignancies.^[9]^ Gallbladder carcinosarcoma lacks specific clinical manifestations, imaging features, serum biochemistry patterns, or characteristic tumor markers. Most patients present at an advanced stage, leading to a poor prognosis. Pathology and immunohistochemistry serve as the diagnostic gold standard. Treatment is primarily surgical, with the role of adjuvant radiotherapy still unclear and under investigation.^[10]^ The most common sites of postoperative metastasis are the liver and peritoneum, although spread to the adrenal glands, pancreas, and lower thoracic vertebrae can also occur.^[8]^ Consistent with Case 2, our patient developed peritoneal metastasis 6 months postoperatively. Following a change in treatment regimen, subsequent CT and PET-CT scans indicated significant improvement.

The early peritoneal recurrence in Case 2, occurring within 6 months of an R0 resection with negative lymph nodes, raises the important question of whether microscopic peritoneal dissemination or occult nodal disease may have been present at the time of initial surgery. Several mechanisms could explain this phenomenon. First, gallbladder carcinosarcoma is known for its aggressive biological behavior and high propensity for early metastasis, even when the primary tumor appears localized.^[7,9]^ Second, the gallbladder lacks a serosal layer on its hepatic side, which may facilitate direct tumor cell seeding into the peritoneal cavity or via lymphatic channels not adequately sampled during routine lymphadenectomy.^[11]^ Third, standard histopathological examination may miss isolated tumor cells or micrometastases in lymph nodes or peritoneal surfaces, particularly when only a limited number of nodes are examined. In Case 2, only 6 lymph nodes were retrieved, which may be insufficient to fully exclude nodal involvement. Occult peritoneal disease, undetectable by preoperative imaging or intraoperative inspection, could also have been present and later progressed to clinically evident metastases under the influence of postoperative immunosuppression or surgical manipulation. These considerations highlight the inherent limitations of current staging modalities in predicting outcomes for highly aggressive tumor subtypes and support the need for more sensitive detection methods, such as peritoneal cytology or molecular staging, in future studies.

Both patients achieved R0 resection and had negative lymph nodes. However, the number of lymph nodes retrieved was limited (4 in Case 1, 6 in Case 2). Adequate lymph node sampling is critical for accurate staging in gallbladder cancer, as nodal status is a key prognostic factor.^[11]^ A low lymph node yield may underestimate nodal disease and potentially contribute to understaging. Regarding treatment, radical cholecystectomy remains the primary approach, with a negative surgical margin widely believed to potentially reduce the risk of postoperative metastasis.^[12,13]^ Relevant literature indicates that prognosis is better for patients undergoing radical surgery compared to palliative surgery, and for those with negative margins compared to positive margins.^[2]^ However, even with negative margins, recurrence and metastasis can occur rapidly, most commonly in the liver. This may be due to the lack of a serosal layer between the gallbladder and liver, facilitating spread.^[11]^

Postoperative adjuvant chemotherapy was administered in both cases following current guidelines. The NCCN guidelines recommend postoperative adjuvant chemotherapy with regimens such as capecitabine monotherapy, capecitabine combined with cisplatin/oxaliplatin, gemcitabine monotherapy, gemcitabine combined with cisplatin/capecitabine, or 5-fluorouracil monotherapy or combined with cisplatin/oxaliplatin.^[14]^ In Case 1, 2 cycles of gemcitabine plus capecitabine were given; in Case 2, 3 cycles of gemcitabine monotherapy were used, consistent with NCCN and Chinese guideline recommendations for high-risk gallbladder cancer (≥T2, node-negative, R0 resection).^[10,11]^ After recurrence, Case 2 received a combination of toripalimab (anti-PD-1), oxaliplatin, lenvatinib, and capecitabine. This regimen was chosen as an individualized salvage therapy based on emerging evidence supporting immunotherapy in biliary tract cancers^[15]^ and the patient’s rapidly progressive disease. It is important to note that no molecular testing (e.g., microsatellite instability, PD-L1 expression, tumor mutational burden) was performed to guide this choice, representing a limitation. While the radiologic response was encouraging, this approach remains exploratory and outside current standard-of-care recommendations.

In recent years, immunotherapy has shown breakthrough progress in advanced biliary tract cancer, with immune checkpoint inhibitor combination chemotherapy becoming a first-line standard. Relevant studies indicate that adding immunotherapy to gemcitabine and cisplatin significantly improves overall survival and 18- and 24-month survival rates compared to chemotherapy alone.^[15]^ Although its value in the adjuvant setting awaits confirmation by high-level evidence, for patients with rapid postoperative recurrence or metastasis, adopting advanced disease treatment strategies – combining immune checkpoint inhibitors with chemotherapy and/or targeted agents (e.g., toripalimab + oxaliplatin + capecitabine + lenvatinib in this case) – has demonstrated encouraging efficacy. This suggests that future guideline updates should consider discussing more individualized adjuvant strategies incorporating immunotherapy for rare subtypes or high-risk populations. The clinical behavior of our cases aligns with previous reports on rare gallbladder malignancies. Sarcomatoid carcinoma of the gallbladder often presents at an advanced stage and is associated with early liver metastasis and poor survival.^[2,3,14]^ The relatively favorable outcome in Case 1 (no recurrence at 6 months) may reflect early stage (T1b) and R0 resection. In contrast, carcinosarcoma is recognized as one of the most aggressive biliary tract tumors, with high rates of peritoneal and hepatic metastasis even after curative resection.^[7,9]^ Teng et al reported a median survival of only 7 months for gallbladder carcinosarcoma, with 1-year and 5-year survival rates of 19% and 16%, respectively.^[9]^ The rapid peritoneal recurrence in Case 2 is consistent with this aggressive biology and underscores the need for close surveillance and early intervention.

Both patients with rare pathological subtypes in our ongoing follow-up are alive. Their pathologies were gallbladder carcinosarcoma and sarcomatoid carcinoma, respectively. Both achieved R0 resection and received varying cycles of postoperative chemotherapy. The patient with carcinosarcoma unexpectedly developed metastasis at 6 months. After 4 cycles of Toripalimab-based therapy, follow-up imaging showed significant reduction. As of the last follow-up, this patient had completed 7 cycles of toripalimab (240 mg) + oxaliplatin (150 mg) + oral lenvatinib (8 mg) + oral capecitabine (1.5 g), with stable vital signs, good general condition, and no reported adverse reactions, indicating the effectiveness of the regimen. Given the low survival rates associated with these tumors – literature reports 1-year and 5-year survival rates of approximately (19 ± 5)% and (16 ± 5)%, respectively, with the longest survival being 54 months^[9]^ – vigilant follow-up and timely treatment modification upon recurrence or metastasis are crucial.

In summary, gallbladder malignancies, due to their lack of characteristic features and specific tumor markers, are often diagnosed at an advanced stage, resulting in poor prognosis and low 5-year survival rates. The primary treatment is surgical, namely radical cholecystectomy. A negative surgical margin is believed to potentially reduce the risk of metastasis. However, even with this, metastasis can occur rapidly, as evidenced by Case 2 developing peritoneal metastasis at 6 months. The liver is the most common metastatic site, likely due to the absence of a serosal layer between the gallbladder and liver.^[11]^ Both gallbladder carcinosarcoma and sarcomatoid carcinoma are extremely rare pathological subtypes, with pathology and immunohistochemistry being the diagnostic gold standards. The cases presented here highlight the diagnostic challenges posed by atypical immunohistochemical profiles, the potential limitations of standard staging in predicting aggressive behavior, and the emerging role of immunotherapy-based regimens in salvage settings for rare aggressive subtypes.

## 4. Conclusion

We believe that upon detection of gallbladder cancer, patients should undergo timely surgical intervention, specifically radical cholecystectomy. Early-stage patients may be candidates for simple cholecystectomy, while radical cholecystectomy with partial hepatectomy (segments IVb/V) and lymph node dissection is more strongly recommended for intermediate and advanced stages. Achieving an R0 resection is a key prognostic factor. These 2 cases of rare gallbladder malignancies provide several important lessons. First, the diagnosis of sarcomatoid carcinoma can be challenging when cytokeratin expression is lost; a comprehensive immunohistochemical panel including epithelial and mesenchymal markers is essential to distinguish it from pure sarcomas. Second, even after R0 resection and negative lymph nodes, aggressive subtypes such as carcinosarcoma may recur early, indicating that standard staging may underestimate their biologic behavior. Third, in the setting of rapid postoperative recurrence, immunotherapy-based combination regimens may offer a salvage option, although their role in adjuvant settings requires further validation. These cases underscore the need for heightened awareness, meticulous surgical technique, and individualized surveillance strategies for rare pathological types of gallbladder cancer.

## Author contributions

**Conceptualization:** Zepu Wang.

**Investigation:** Yang Zhao, Yaqi Xiang.

**Methodology:** Jun Li.

**Supervision:** Hongfang Tuo.

**Writing – original draft:** Hongfang Tuo.

**Writing – review & editing:** Hao Wang.

## Correction

The online version has now been updated by linking the 2nd affiliation "Department of Hepatobiliary and Pancreatic Surgery, Hebei General Hospital, Shijiazhuang, Hebei, China" to the Authors "Hao Wang, Yang Zhao, Yaqi Xiang, Jun Li, Hongfang Tuo."
